# Does Early Laser Treatment of Capillary Malformations Lead to More Favorable Outcomes?

**DOI:** 10.1002/lary.70099

**Published:** 2025-09-03

**Authors:** Larkin Harris, Reema Padia

**Affiliations:** ^1^ Department of Otolaryngology‐Head and Neck Surgery University of Utah Salt Lake City Utah USA

**Keywords:** aesthetic surgery, pediatric general, pediatrics, skin resurfacing modalities: Chemical peel, dermabrasion, laser, vascular anomalies

## 1 Background

Capillary malformations (CMs) or port‐wine stains are low‐flow congenital vascular anomalies impacting approximately three children per 1000 births. CMs most commonly occur on the face and may be associated with genetic syndromes such as Sturge‐Weber Syndrome (SWS) [1]. Depending on their location, CMs may result in psychosocial distress, dysphagia, dysphonia, and airway obstruction. In the setting of SWS, airway obstruction and exacerbation of obstructive sleep apnea may also contribute to worsening of neurologic outcomes [1, 2]. The natural progression of untreated CMs can include darkening, increased risk of bleeding, nodularity, and dermal hypertrophy of the involved tissue potentially impacting deglutition, airway status, and vision [1].

The gold standard in the treatment of CMs is laser therapy using a pulsed‐dye laser (PDL) [1]. The use of PDL in treating CMs in infants is well documented; however, treatment regimens vary significantly. In recent years, there has been debate regarding the optimal timing of treatment for CMs due to the potential impact of anesthetic agents in children. These concerns derive from animal models indicating potential neuronal apoptosis and disruption of neurodevelopment; although, human data remain inconclusive [3]. When treating CMs, physicians must weigh the benefits of early treatment versus the potential harm of anesthetic agents.

## 2 Literature Review

The use of the pulsed‐dye laser (PDL) has been described in the treatment of CM since the early 1990s. PDL effectively treats CMs by emitting photons that are absorbed by hemoglobin, inducing thermal denaturation, coagulation, and inflammation, which in turn stimulates vascular remodeling and angiogenesis of normal‐sized blood vessels with resultant lightening of lesions [4].

It has been previously suggested that initiating PDL therapy in children less than 1 year of age may halt lesion progression, reduce the risk of recurrence, and have a higher likelihood of clearance. Multiple mechanisms have been suggested for the superior response seen in infants undergoing PDL treatment for CMs. Younger patients have smaller blood vessels, a thinner dermis, and fewer circulating red blood cells, potentially resulting in a more targeted and efficacious response to laser therapy. Treatment response to PDL is inversely correlated with vascular depth, epidermal thickness, and stratum corneum thickness, which are optimized in infancy [4]. Additionally, early treatment can halt the progression of CMs, limiting downstream sequelae such as disfigurement, vision changes, or bleeding. In 2014, a consensus statement recommended against repetitive elective general anesthesia in infants and toddlers, giving physicians pause with early initiation of treatment [3].

PDL therapy in infants can be performed with local analgesia without the need for a general anesthetic. Topical analgesics such as cryogens or a eutectic mixture of lignocaine and prilocaine (EMLA) are most commonly used. PDL treatments, especially on the face, can be uncomfortable, and the adequacy of analgesia is difficult to assess in infants. Larger lesions will require more pulses, and sensitive areas of the face such as the eyelids require the placement of protective eye shields, further exacerbating pain. These factors should be considered to minimize patient trauma and optimize plans for subsequent treatments. Pain experienced in infancy is not benign and may be associated with lasting differences in pain perception among other unknown psychosocial consequences.

Studies suggest that the most significant improvement occurs during the first 5 treatments. Infants with lesions less than 20 cm [1] overlying bony areas such as the central forehead have achieved complete clearance within five treatments [4].

The intervals used in PDL treatments for CMs vary in the literature from 7 days to 8 weeks. Previous work suggests that shorter treatment intervals may be more efficacious and reduce the overall duration of treatment. There is evidence that the expression of proangiogenic genes such as vascular endothelial growth factor peaks 7 days after PDL therapy, potentially stymieing the treatment effect when longer intervals are used. A small retrospective dataset demonstrated treatment intervals with a median of 7 days to be both safe and effective without the development of additional adverse events such as dyschromia [4]. This regimen potentially takes advantage of delayed proangiogenic gene expression and decreased vessel repair in the interim between treatments.

Left untreated, CMs are known to undergo ectasia with darkening, dermal hypertrophy, and the development of nodularity. This growth generally occurs in late childhood or early adolescence [5]. Hypertrophied lesions can be associated with bleeding, vision changes, airway obstruction, development of pyogenic granulomas, disfigurement, and increased risk of psychological harm. Longstanding CMs are less likely to respond to PDL treatment and are more likely to require surgical intervention, which is beyond the scope of this article.

It should be noted that CM is thought to result from activating mutations in specific cell signaling pathways. The most implicated mutations include the *GNAQ* and *GNA11* genes; although, there are other less common mutations that can result in CMs, including *PIK3CA*. As this is a genetic condition, patients may have darkening and progressive nodularity and hypertrophy, which can occur years later despite early and effective treatment, especially in those with a *PIK3CA* variant.

## 3 Best Practice Summary

The treatment of CMs using PDL therapy requires shared decision‐making with the patient and their family. PDL therapy initiated in infancy is associated with better outcomes but should be limited to patients with lesions amenable to topical anesthesia unless airway or eye involvement is present. Early treatment of CMs with PDL may halt disease progression and limit ectasia and hypertrophy in lesions that cannot be completely cleared. Early evidence suggests that minimizing the duration between treatments may result in improved outcomes related to decreased expression of proangiogenic genes. In infants with CMs not amenable to topical analgesia, the risks and benefits of general anesthesia should be considered against the risk of lesion progression and lasting physiological and psychological sequelae. Further research is needed to differentiate best practice among patients of various Fitzpatrick scores. It is known that complications such as dyschromia are more likely to occur in patients with a higher Fitzpatrick score; therefore, this should be considered when making the ultimate decision to proceed with treatment.

## 4 Level of Evidence

This best practice recommendation is based on Level III–V evidence, including expert opinion, retrospective studies, and systematic review data.

## Conflicts of Interest

The authors declare no conflicts of interest.
